# Morphological Evaluation of the Hip Joint and Pelvic Limb Bones of the Lowland Tapir ( *Tapirus terrestris* Linnaeus, 1758) Using Radiography and Computed Tomography

**DOI:** 10.1111/ahe.70110

**Published:** 2026-04-07

**Authors:** Heloísa Coppini de Lima, Sheila Canevese Rahal, Jeana Pereira da Silva, Rodrigo Hidalgo Friciello Teixeira, Maria Jaqueline Mamprim, Bruno Critelli Carvalho, Miriam Harumi Tsunemi

**Affiliations:** ^1^ Graduate Program in Wild Animals, School of Veterinary Medicine and Animal Science São Paulo State University São Paulo Brazil; ^2^ Department of Veterinary Surgery and Animal Reproduction, School of Veterinary Medicine and Animal Science São Paulo State University São Paulo Brazil; ^3^ Municipal Zoological Park “Quinzinho de Barros” São Paulo Brazil; ^4^ Prime Centro Diagnóstico Veterinário São Paulo Brazil; ^5^ Department of Biodiversity and Biostatistics Institute of Biosciences São Paulo Brazil

**Keywords:** angular measurements, hindlimb anatomy, osteology, Perissodactyla

## Abstract

The study aimed to characterize, through imaging examinations—including radiographs and computed tomography—the hip joint and the bones of the thigh, leg and feet in Lowland tapirs ( *Tapirus terrestris* ). Ten pelvic limbs were collected, two from juveniles and the remaining from adults. The femoral head exhibited a rounded morphology with a well‐defined fovea capitis and a short, thick neck. Three trochanters were identified, with the greater and third trochanters being particularly prominent. The femoral diaphysis was elongated and straight, and the distal end displayed a globular medial condyle and a flatter lateral condyle. The femoral trochlea consisted of medial and lateral trochlear crests of similar size, separated by a well‐defined trochlear groove. The proximal tibia showed slightly concave tibial plateaus, with the lateral condyle being wider and a prominent intercondylar eminence. The fibula was positioned laterally along the entire tibia, with the interosseous space preserved. The talus presented a well‐defined trochlea and a head articulating with the central tarsal bone. Tarsal bones I, III and IV were identified, with tarsal IV being elongated. Three metatarsals (II, III and IV) were observed, with metatarsal III relatively more developed. The third digit was larger than both the second and fourth digits. The Norberg angle, femoral head–neck inclination angle, mechanical lateral distal and proximal femoral angles, and anatomical medial proximal and lateral distal tibial angles were similar between the two imaging methods. In conclusion, the imaging examinations were complementary, enabling the characterization of morphological features reflecting the species' functional adaptations for cursorial and semi‐aquatic locomotion.

## 1 Introduction

Tapirs belong to the order *Perissodactyla*, family *Tapiridae* and genus *Tapirus* (Mangini 2014). Considered among the largest terrestrial mammals in their respective ecosystems, tapirs are classified into four species: *Tapirus indicus* , *T. bairdii* , *T. terrestris* and *T. pinchaque* (Sekiama et al. 2011; Mangini 2014). *T. terrestris* , native to South America, is the third‐largest species among them (Mangini 2014; Quse and Fernandes‐Santos 2014).

These animals occupy diverse habitats, including open fields, tropical and deciduous rainforests, dry forests, cerrado and wetlands, and they can even be found near urban areas (Mangini 2014). As herbivores with diets based on fruits and plants, tapirs play a crucial role in seed dispersal—both large and small—since many seeds pass through their digestive system intact and are excreted in their faeces (Galetti et al. 2001; O'Farrill et al. 2013; Mangini 2014). However, tapirs have been severely affected by illegal hunting, deforestation, agricultural expansion, diseases transmitted by domestic animals and roadkill, and their long gestation period further hinders population recovery, slowing the repopulation of affected areas (Mangini 2014).

Physically, tapirs are robust animals of considerable size compared to other Neotropical mammals, with body masses ranging from 150 to 300 kg—values that can be exceeded in the case of the Malayan tapir (Mangini 2014). Females tend to be larger than males, although they do not exhibit pronounced sexual dimorphism (Mangini 2014; Quse and Fernandes‐Santos 2014).

Tapirs are classified as cursorial digitigrades, moving by supporting themselves on their toes (Pereira et al. 2018). As ungulates, their digits are covered by keratinized hooves (Quse and Fernandes‐Santos 2014). Among other skeletal characteristics, tapirs are notable for their short, slender legs and mesaxonic feet, in which the body weight is concentrated mainly on the central digit, with four toes on the forelimbs (tetradactyl) and three on the hindlimbs (tridactyl), adaptations that facilitate locomotion on soft or muddy ground (Padilla and Dowler 1994; Medici et al. 2001; Quse and Fernandes‐Santos 2014).

Although they cannot jump, tapirs can climb fences or walls less than 1.5 m high (Medici et al. 2001). When moving, they keep their heads down while walking slowly and upright while trotting, and they are capable of galloping and climbing slopes (Padilla and Dowler 1994). In the forest, they can run at speeds comparable to those of a human and are excellent swimmers, often seeking refuge in the water (Padilla and Dowler 1994; Medici et al. 2001; Feldhamer et al. 2015).

Although studies have addressed the anatomical aspects of the hind limbs of tapirs (Borges et al. 2018), research using imaging exams is still limited (Endo et al. 2019; Borges et al. 2021; Costa et al. 2022). Such exams can provide complementary information and contribute to a better understanding of normal patterns in the species.

Thus, the aim of this study was to characterize, through imaging examinations—including radiography and computed tomography (CT)—the features of the hip joint and the bones of the thigh, leg and feet in Lowland tapirs ( *Tapirus terrestris* ).

## 2 Materials and Methods

### 2.1 Specimen Collection and Imaging Examinations

This study was approved by the Institutional Ethics Committee on the Use of Animals (CEUA, no. 000.030). Ten pelvic limbs from *T. terrestris* were collected, including four limbs from two juveniles (aged 3–6 months) and six limbs from adult specimens (older than 3 years). The specimens were preserved either frozen (*n* = 6; two limbs from a juvenile and four from adult individuals) or in salt (*n* = 4; two limbs from a juvenile and two from adult individuals) until the imaging examinations were performed.

For imaging procedures, frozen specimens were thawed at room temperature. Radiographic examination of the hip joint in juveniles was performed using a ventrodorsal projection with the limbs extended caudally (70 kVp and 8 mAs). For the thigh and leg bones, craniocaudal and mediolateral projections were obtained (60–80 kVp and 8 mAs), while dorsoplantar and mediolateral projections were used for the feet (65 kVp and 8 mAs). For all examinations, a focus‐to‐film distance of 100 cm was applied. A digital radiography system was used (DR‐F, GE Health Care Unit, Chicago, IL, USA). Images were stored in DICOM (Digital Imaging and Communications in Medicine) format and analysed using the RadiAnt DICOM Viewer software (Medixant, Poznań, Poland).

CT examinations were performed using either a 16‐slice Aquilion Start scanner (Canon Medical Systems; 120 kVp, 70 mA, slice thickness of 1 mm) or an 80‐slice Aquilion Serve scanner (Canon Medical Systems; 120 kVp, 150 mA, slice thickness of 0.5–1 mm). The acquired images were subjected to multiplanar reconstruction (MPR) and three‐dimensional (3D) reconstruction.

Based on the images obtained, the radiographic and/or tomographic anatomy of the thigh bones (femur), leg (tibia) and feet (tarsus, metatarsus and phalanges) was described. Measurements were also performed using ventrodorsal/craniocaudal radiographs and CT scans, employing MPR images in the dorsal plane, including the Norberg angle, femoral head and neck inclination angle (Hauptman B) and femoral mechanical angles. In the tibia, anatomical angles were analysed using craniocaudal radiographs and MPR images in the dorsal plane. All measurements were performed in triplicate by a researcher experienced in imaging analysis.

### 2.2 Angle Measurements

#### 2.2.1 Norberg Angle

Both juvenile specimens were used for determination of the Norberg angle (Petazzoni and Jaeger 2008). This measurement could only be assessed in the juvenile specimens, as they were the only individuals in which the hip bone was preserved. The same radiographic methodology was adapted for CT. Initially, the centres of the femoral heads were identified by fitting circles to each head. A straight line connecting the centres of the femoral heads was then drawn. From the centre of each femoral head, a second line was traced towards the dorsocranial margins of the right and left acetabula, and the angle formed by these lines corresponded to the respective Norberg angles.

#### 2.2.2 Femoral Head and Neck Inclination Angle

All juveniles (four limbs) and adults (four limbs) were included in the evaluation of the femoral inclination angle using Hauptman's method B (Hauptman et al. 1979). This method consisted of bisecting the femoral diaphysis along its longitudinal axis. Subsequently, the femoral neck was transversely sectioned at its narrowest point. A line connecting the central points of the femoral head and femoral neck was then drawn. The angle formed between this line and the diaphyseal axis corresponded to the femoral inclination angle.

#### 2.2.3 Femoral Mechanical Angles

All juveniles (four limbs) and adults (four limbs) were used for determination of the femoral mechanical angles, as described by Tomlinson et al. (2007). Initially, the femoral mechanical axis was established as a line connecting the centre of the femoral head to the most proximal point of the intercondylar fossa. Subsequently, the distal joint reference line was drawn, and its intersection with the mechanical axis defined the mechanical lateral distal femoral angle (mLDFA).

Thereafter, a line was drawn from the centre of the femoral head to the most proximal point of the greater trochanter. The intersection of this line with the mechanical axis defined the mechanical lateral proximal femoral angle (mLPFA).

#### 2.2.4 Tibial Anatomical Angles

All juveniles (four limbs) and adults (six limbs) were used for determination of the tibial anatomical angles. The anatomical axes were defined as two distinct straight lines along the mid‐diaphysis of the tibia, representing the proximal and distal portions of the bone. For this purpose, the tibial diaphysis was divided into two segments of equal length. In each segment, a straight line was drawn through the mid‐diaphysis using points located at 25% and 75% of the segment length, serving as the basis for determination of the respective anatomical axes.

The proximal joint reference line was established by connecting two points on the concave surface of the tibial plateau. Similarly, the distal joint reference line was drawn by connecting two reference points located in the subchondral bone of the grooves of the tibial cochlea. The joint orientation angles were then calculated from the intersection of these lines, namely the anatomical medial proximal tibial angle (aMPTA) and the anatomical lateral distal tibial angle (aLDTA), corresponding to the proximal and distal orientations, respectively (Hette et al. 2009).

### 2.3 Statistical Analysis

To evaluate the data from radiographic and CT measurements, the non‐parametric Wilcoxon test was applied, as the Shapiro–Wilk test indicated that the variables did not follow a normal distribution. Comparisons of median values between juveniles and adults were conducted using the non‐parametric Mann–Whitney test, performed separately for each limb. Statistical analyses were carried out using Microsoft Excel and the R software (R Core Team, 2025). Differences were considered statistically significant at *p* < 0.05.

## 3 Results

**FIGURE 1:**
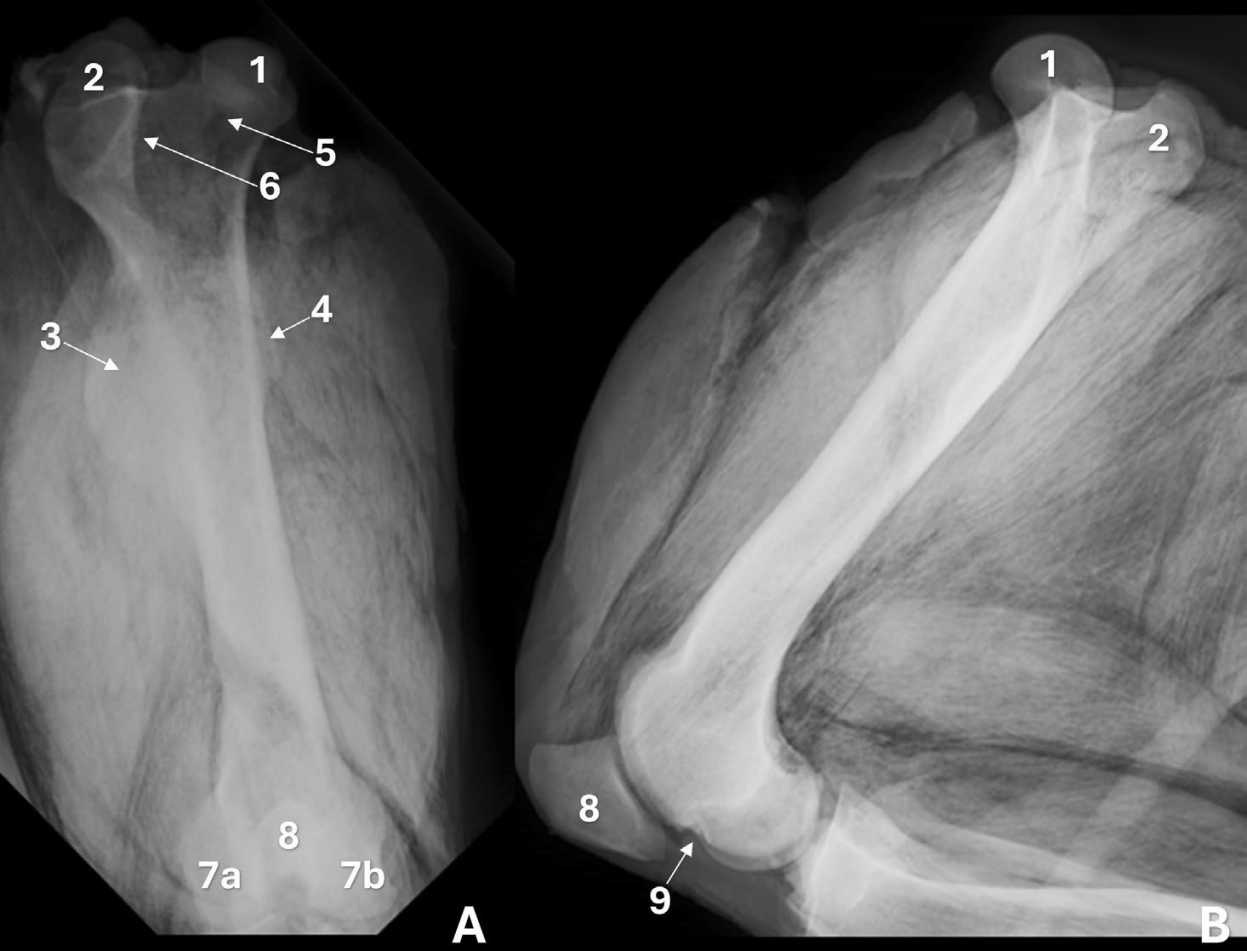
Radiographic images of the femur of an adult Lowland tapir in craniocaudal (A) and mediolateral (B) views. 1: femoral head; 2: greater trochanter; 3: third trochanter; 4: lesser trochanter; 5: femoral neck; 6: trochanteric fossa; 7a: medial condyle; 7b: lateral condyle; 8: patella; 9: extensor fossa.

**FIGURE 2:**
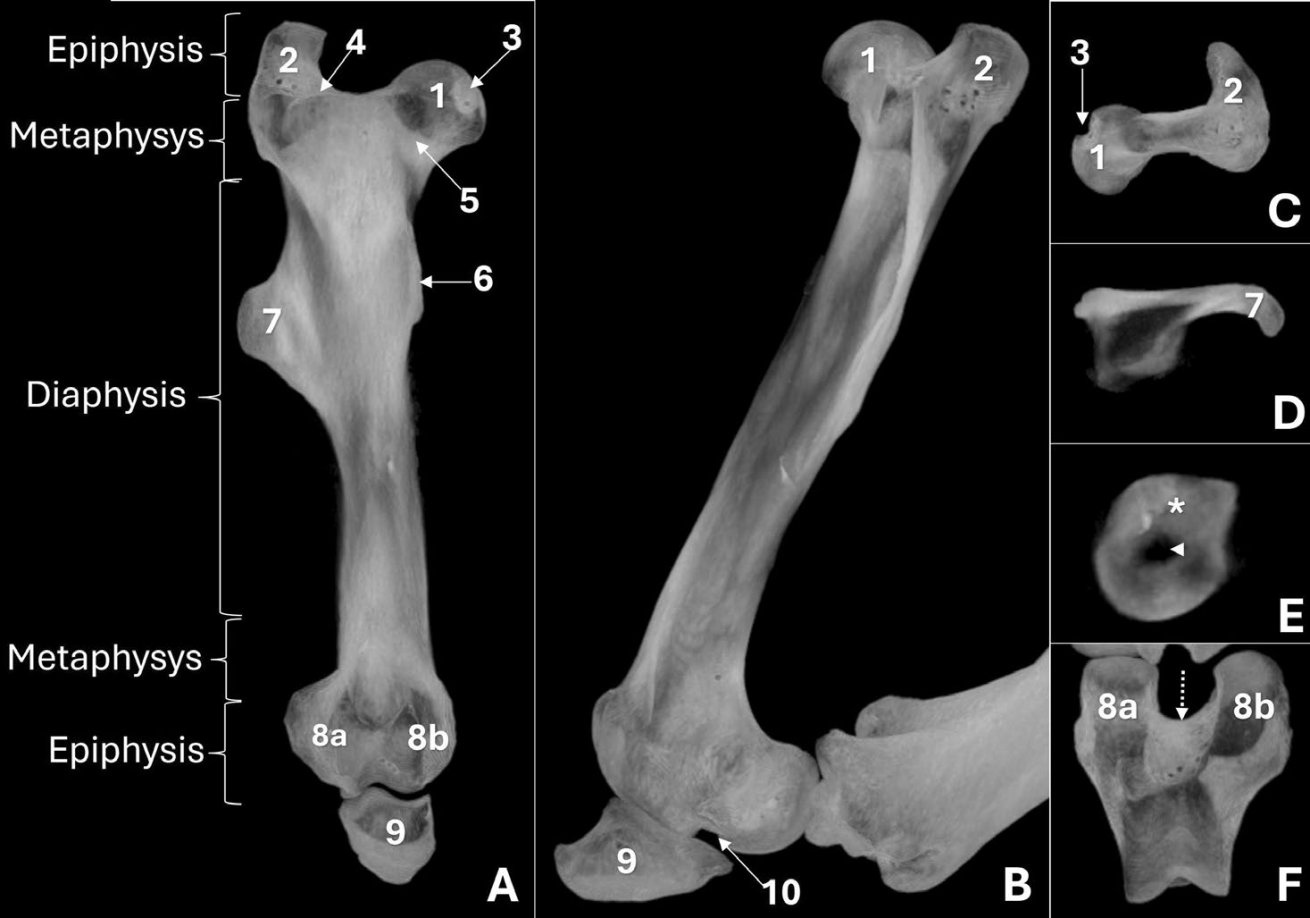
Computed tomographic images of the femur of an adult Lowland Tapir in dorsal (A) and sagittal (B) maximum intensity projection (MIP) reconstructions, and transverse MIP sections of the femoral epiphysis (C), proximal diaphysis (D), mid‐distal diaphysis (E) and distal epiphysis (F). 1: femoral head; 2: greater trochanter; 3: fovea capitis; 4: trochanteric fossa; 5: femoral neck; 6: lesser trochanter; 7: third trochanter; 8a: lateral condyle; 8b: medial condyles; 9: patella; 10: extensor fossa. The asterisk indicates cortical thickness, and the arrowhead indicates the medullary cavity in the mid‐diaphyseal region (E). The dotted arrow indicates the trochlear groove (F).

**FIGURE 3:**
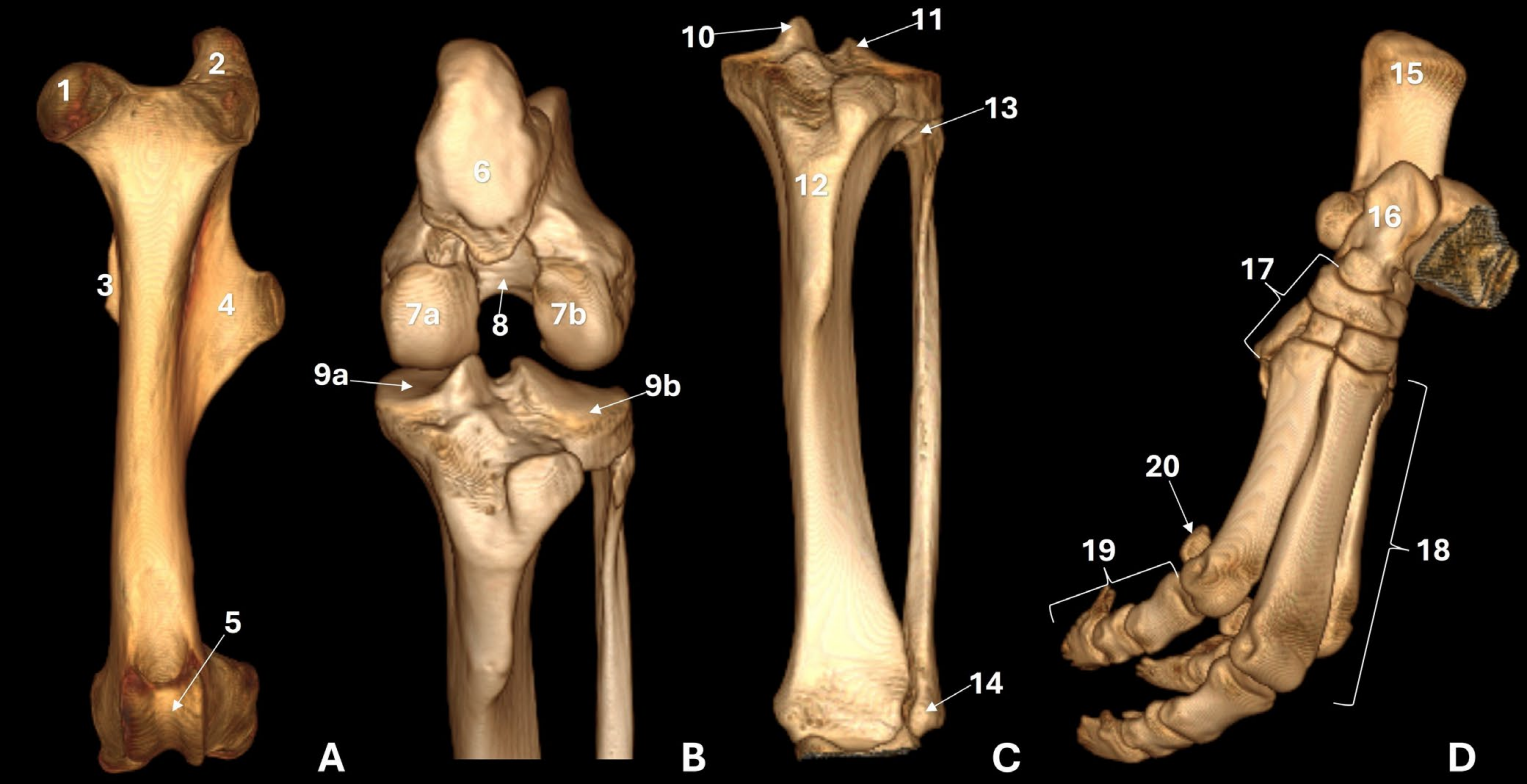
Three‐dimensional reconstructions of the femur (A), distal femur and proximal tibia (B) and tibia/fibula (C) in cranial view; tarsal and metatarsal bones and phalanges (D) in craniomedial view of an adult tapir. 1: femoral head; 2: greater trochanter; 3: lesser trochanter; 4: third trochanter; 5: femoral trochlea; 6: patella; 7a: medial condyle; 7b: lateral condyle; 8: intercondylar fossa; 9a: lateral condyle; 9b: medial condyle; 10: medial intercondylar tubercle; 11: lateral intercondylar tubercle; 12: tibial tuberosity; 13: fibular head; 14: lateral malleolus; 15: calcaneus; 16: talus; 17: tarsal bones; 18: metatarsal bones; 19: phalanges; 20: sesamoid bones.

In specimens preserved in salt, a loss of image quality was observed in both radiographs and CT scans; however, all specimens were still included in the analyses. The femur presented a rounded femoral head with a smooth articular surface (Figure 1A), and the fovea capitis was identified as a pronounced depression on its medial surface (Figure 2A,C). The femoral neck was short and thick (Figure 1A). The femur displayed three distinct trochanters: the greater, lesser and third trochanters (Figure 1A). The greater trochanter projected above the level of the femoral head and was easily recognized on radiographs, particularly in the craniocaudal view (Figure 1A,B). The trochanteric fossa was well defined and separated the greater trochanter from the femoral neck (Figure 1A). The lesser trochanter appeared as a smaller medial protuberance, with clearer delineation on CT due to reduced superimposition (Figure 2A). The third trochanter was well developed, projecting from the lateral aspect of the proximal to middle third of the diaphysis and was well delineated on CT (Figure 2A,D; Figure 3A).

The femoral body exhibited an elongated conformation with a predominantly straight axis (Figure 1; Figure 2A,B). The cortex was continuous and well developed along the diaphysis, showing slight thickening in the middle to distomedial segment and delimiting a centrally positioned medullary canal. Radiography allowed evaluation of the bone axis and overall contour, whereas CT provided better assessment of cortical thickness, bone density and the corticomedullary relationship (Figure 2E). At the distal end, the femur showed progressive widening, forming well‐defined medial and lateral condyles (Figure 1A). Both condyles displayed convex articular surfaces; however, the medial condyle exhibited more pronounced convexity and a more globular appearance, whereas the lateral condyle presented a relatively flatter articular surface and a more elongated conformation (Figure 2F; Figure 3B). The intercondylar fossa was observed between the condyles (Figure 3B). On the caudal aspect of the lateral femoral condyle, a well‐defined and deep depression was identified, corresponding to the groove (fossa) for the popliteus muscle, as well as a second concavity with regular contours related to the extensor fossa (Figure 1B; Figure 2B). On the medial femoral condyle, a concavity with regular contours was also identified, although it was shallower. The craniocaudal radiographic view allowed recognition of both condyles (Figure 1A), whereas overlap in the mediolateral view limited their individualization (Figure 1B). CT enabled detailed characterization of the articular surfaces and caudal concavities (Figure 2; Figure 3).

The patella was located cranial to the femoral distal end and exhibited a triangular to slightly oval morphology with a smooth caudal articular surface (Figure 1B, Figure 2B). The femoral trochlea was composed of medial and lateral trochlear ridges of similar size, separated by a well‐defined trochlear groove with a deep concavity, which was more clearly characterized on CT (Figure 2F; Figure 3A).

**FIGURE 4:**
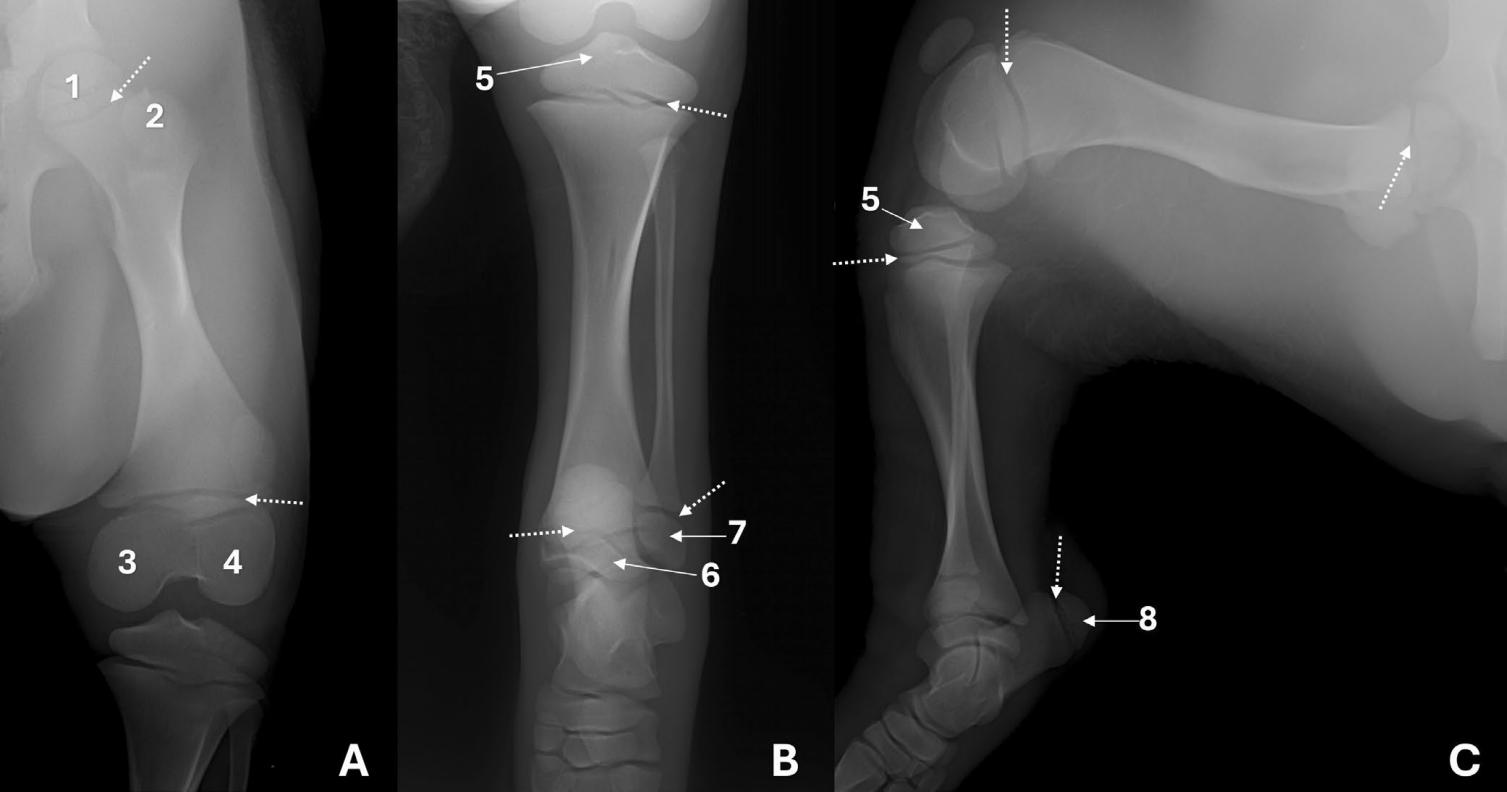
Radiographic images of the femur (A) and tibia (B) in craniocaudal views and the femur and tibia/fibula (C) in mediolateral view of a juvenile tapir. 1: femoral head; 2: greater trochanter; 3: medial condyle; 4: lateral condyle; 5: tibial condyles; 6: distal tibial epiphysis; 7: lateral malleolus; 8: calcaneal tuberosity. Arrows indicate open growth plates.

**FIGURE 5:**
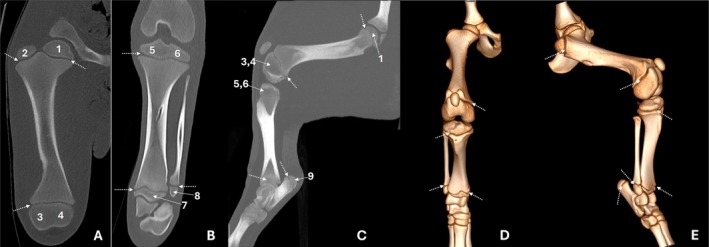
Tomographic images of a juvenile tapir in dorsal maximum intensity projection (MIP) reconstructions of the femur (A) and tibia (B), sagittal MIP of the femur/tibia (C) and three‐dimensional reconstructions in dorsal (D) and dorsolateral (E) views. 1: femoral head; 2: greater trochanter; 3: lateral condyle; 4: medial condyle; 5: medial tibial condyle; 6: lateral tibial condyle; 7: distal tibial epiphysis; 8: lateral malleolus; 9: calcaneal tuberosity. Arrows indicate open growth plates.

In the femur of the juvenile animal, both craniocaudal and mediolateral radiographic examinations, as well as CT evaluation, allowed identification of the growth plates, including the proximal femoral, greater trochanteric and distal femoral physes (Figure 4; Figure 5). The lesser and third trochanters were better visualized on CT.

**FIGURE 6:**
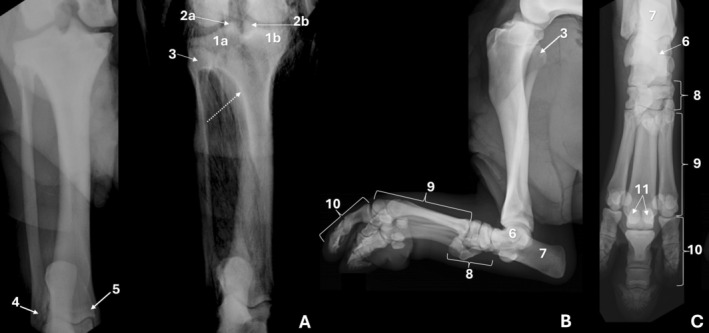
Radiographic images of the tibia/fibula and foot of an adult Lowland Tapir in craniocaudal (A), mediolateral (B) and dorsoplantar (C) views. 1a—lateral condyle; 1b: medial condyle; 2a: lateral intercondylar tubercle; 2b: medial intercondylar tubercle; 3: proximal extremity of the fibula; 4: lateral malleolus; 5: medial malleolus; 6: talus; 7: calcaneus; 8: tarsal bones; 9: metatarsals; 10: phalanges; 11: sesamoid bones.

**FIGURE 7:**
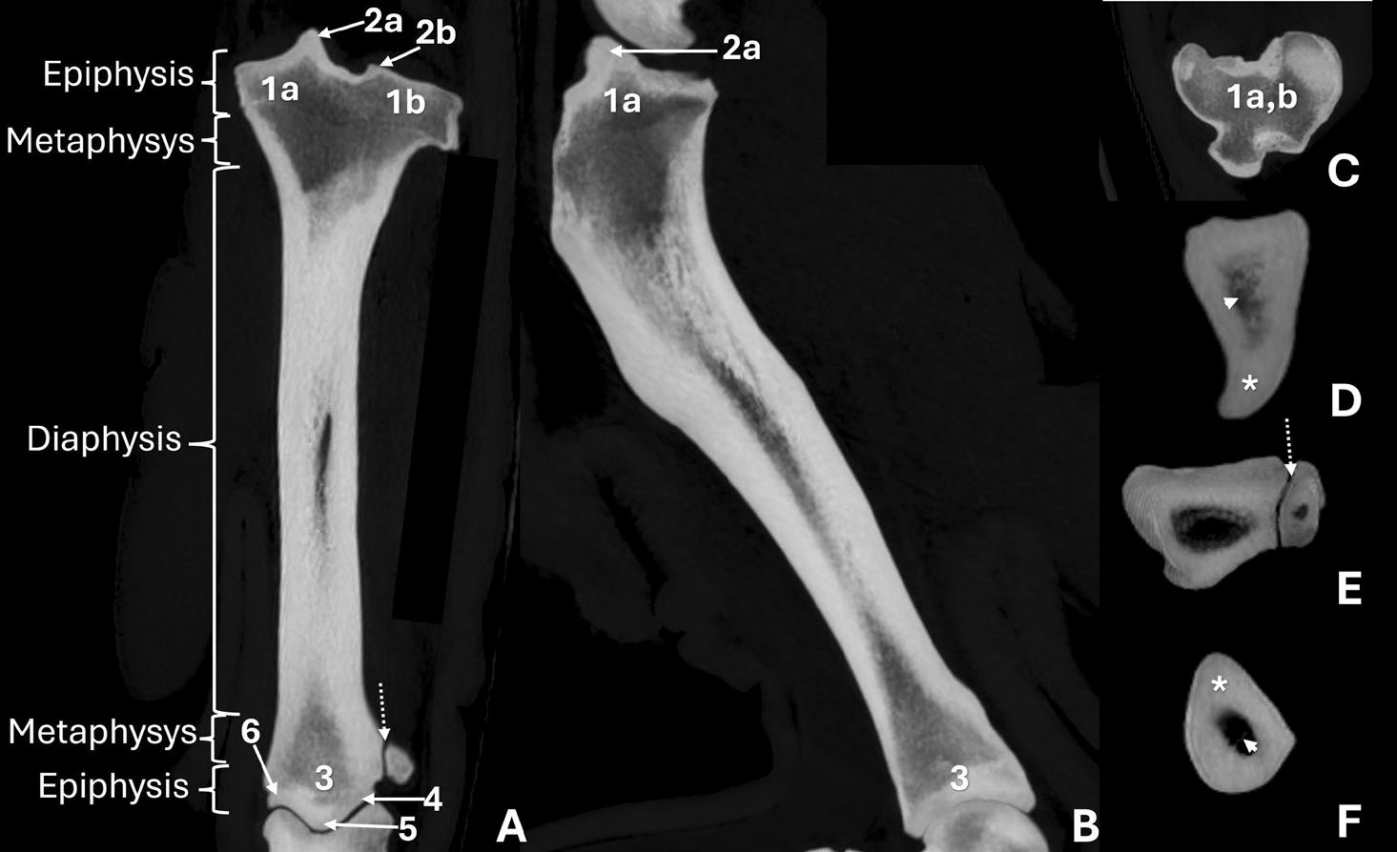
Tomographic images of the adult Lowland Tapir tibia, in dorsal (A) and sagittal (B) maximum intensity projection (MIP) reconstructions and MIP transverse sections of the proximal tibial epiphysis (C), proximal diaphysis (D), mid‐distal diaphysis (E), and distal epiphysis (F). 1a: medial condyle, 1b: lateral condyle; 2a: medial intercondylar tubercle, 2b: lateral intercondylar tubercle; 3: distal end of the tibia; 4: lateral sulcus of the cochlea; 5: intermediate crest of the cochlea; 6: medial malleolus. The dotted arrow indicates the distal tibiofibular joint (A, E). The asterisk shows the cortical thickness, and the arrowhead shows the medullary cavity of the proximal (D) and distal (F) regions of the diaphysis.

The proximal end of the tibia exhibited medial and lateral tibial plateaus with slightly concave articular surfaces, with the lateral condyle appearing wider (Figure 3B). The intercondylar eminence was observed, composed of the medial and lateral intercondylar tubercles, with the medial tubercle being more prominent (Figure 3C). Craniocaudal radiographs (Figure 6A) and dorsal CT images consistently demonstrated the intercondylar region, while CT allowed better definition of the central, cranial and caudal intercondylar areas (Figure 7A–C). The tibial tuberosity was identified on the cranial aspect of the proximal end, with superior characterization on CT (Figure 3C). The craniocaudal radiographic examination identified an opacity resulting from border overlap (Figure 6A). The tibia exhibited a long, prismatic diaphysis with a thick, regular and continuous cortex and a well‐defined medullary canal. On radiography, the bone axis appeared straight in the craniocaudal view, whereas a slight curvature was observed in the mediolateral view (Figure 6A,B). CT allowed more precise evaluation of cortical thickness, medullary canal symmetry and bone density along the diaphysis (Figure 7).

At the distal end, the tibia showed progressive widening and a well‐defined articular surface, with formation of the medial malleolus (Figure 6A). Craniocaudal and mediolateral radiographs allowed evaluation of the bone shaft, overall contour and the tibiotarsal and distal tibiofibular joints, enabling assessment of articular congruency, although fine details were limited by superimposition (Figure 6A,B). CT imaging provided superior visualization of the articular morphology, clearly demonstrating the tibial cochlea (trochlea) with medial and lateral grooves separated by an intermediate crest, as well as more precise delineation of the fibular notch and its relationship with the fibula (Figure 3C; Figure 7A,E).

The fibula appeared as a slender bone located lateral to the tibia (Figure 3C; Figure 6A). Proximally, the fibular head was identified in relation to the caudolateral aspect of the lateral tibial condyle, followed by a narrow neck. The fibular shaft maintained a continuous cortex and a narrow medullary canal, remaining separated from the tibia by a distinct interosseous space along its length. Proximal and distal tibiofibular relationships were observed without evidence of fusion between the bones (Figure 3C). Distally, the fibula lay in close apposition to the fibular notch of the tibia, constituting the lateral malleolus. Radiographs allowed assessment of overall alignment and the general tibiofibular relationship (Figure 6A,B), whereas CT confirmed the absence of fusion (Figure 3C; Figure 7A,E).

The growth plates identified in the juvenile animal were the proximal tibial, distal tibial, medial malleolar and distal fibular growth plates (Figure 4B,C; Figure 5B–5E). The growth plates of the tibial tuberosity and the proximal fibula could not be identified.

**FIGURE 8:**
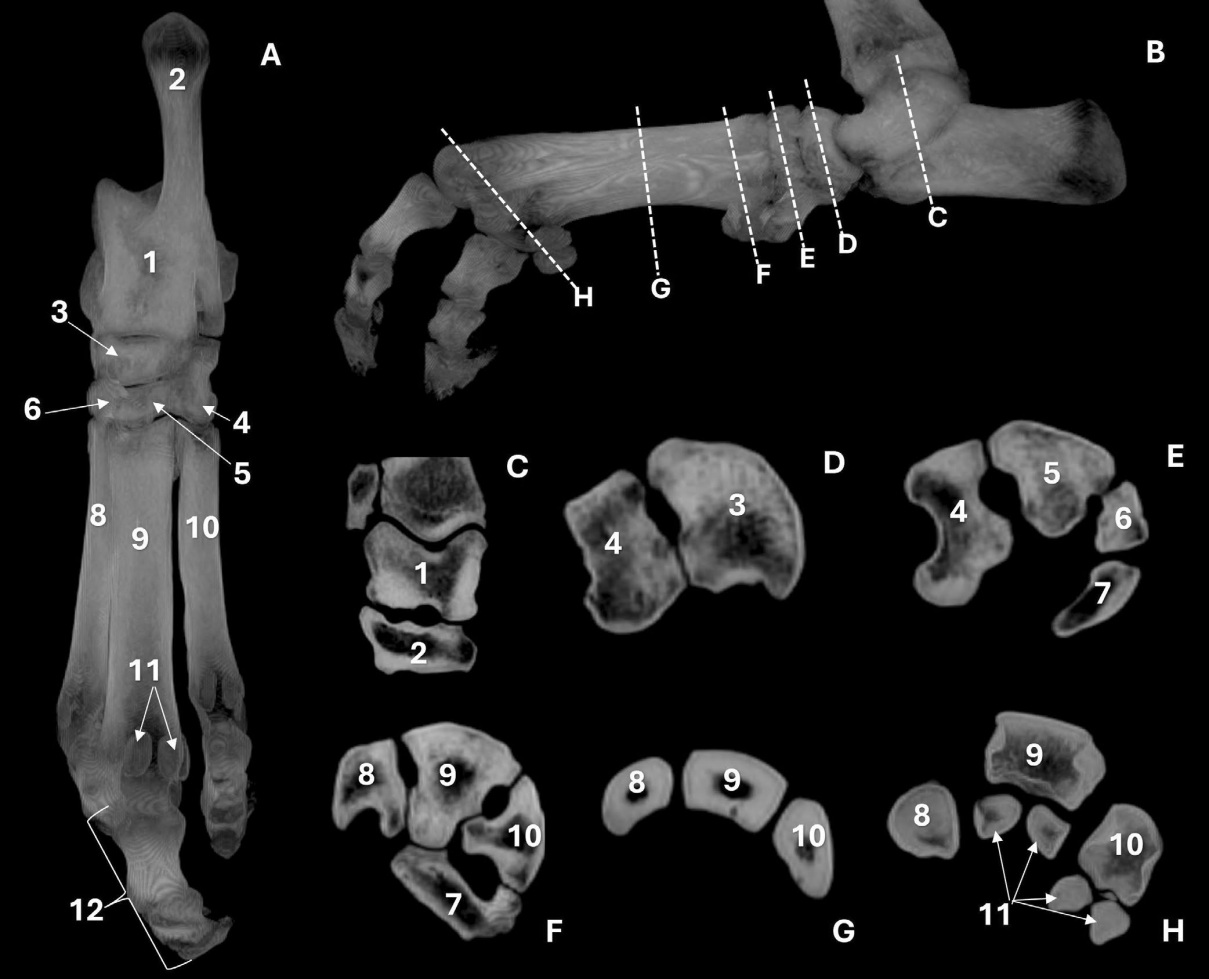
Tomographic images of the tarsus, metatarsus and digits of an adult Lowland Tapir in dorsal (A) and sagittal (B) maximum intensity projection (MIP) reconstructions, and transverse MIP sections at the level of the tarsus (C–E) and the proximal (F), middle (G) and distal (H) metatarsus. 1: talus; 2: calcaneus; 3: central tarsal bone (navicular); 4: tarsal IV; 5: tarsal III; 6: tarsal II; 7: tarsal I; 8: metatarsal IV; 9: metatarsal III; 10: metatarsal II; 11: sesamoid bones; 12: digits.

The talus exhibited a well‐developed body with a clearly defined trochlea, composed of a central trochlear groove and medial and lateral grooves separated by ridges, forming a regular articular surface for the tibiotalar joint (Figure 6B,C). Distally, the neck extended to the head of the talus, whose articular surface for the navicular bone was well defined, forming the talonavicular joint. The calcaneus exhibited an elongated body and a prominent calcaneal tuberosity with evident medial and lateral processes, as well as identification of the coracoid process on CT imaging. The central tarsal bone (navicular) exhibited a prominent plantar tuberosity and articulated distally with the distal tarsal bones via the centrodistal joint (Figure 8A,B,D). Among the distal tarsal bones, tarsals I, III and IV were identified, with tarsal IV being elongated and presenting a well‐defined tuberosity (Figure 6B,C; Figure 8A,B,F,G). It articulated proximally with the calcaneus and distally with the metatarsals, also forming the tarsometatarsal joint. On the CT, in the plantar view, two sesamoid bones were identified at the distal end of each metatarsal bone (Figure 8A,H).

The third digit was the largest, relative to the second (medial) and fourth (lateral) digits (Figure 6C; Figure 8A). Each digit comprised proximal, middle and distal phalanges, with the distal phalanges exhibiting a semilunar shape.

No statistically significant differences were observed between the values obtained from radiographic and CT scans, nor between juvenile and adults. The angular measurement values are presented in Table 1.

**TABLE 1:** Values of radiographic and computed tomography measurements of the Norberg angle, angle of inclination of the femoral head and neck (Hauptman B), mechanical lateral proximal femoral angle (mLPFA), mechanical lateral distal femoral angle (mLDFA), anatomical medial proximal tibial angle (aMPTA) and anatomical lateral distal tibial angle (aLDTA), obtained from the bones of Lowland tapirs ( *Tapirus terrestris* ).

Angles	Number of limbs	Radiographic measurements (°)					
		Mean ± SD	Minimum	Quartile 1	Median	Quartile 3	Maximum
Norberg	4	127.5 ± 1.3	126.6	127.1	127.5	128.0	128.5
Hauptman B	8	136.5 ± 6.9	131.1	132.3	132.7	140.3	147.7
mLPFA	8	98.9 ± 14.8	80.0	85.8	105.7	110.3	110.7
mLDFA	8	95.3 ± 3.1	92.4	92.6	94.5	97.8	99.4
aMPTA	10	92.2 ± 1.7	90.5	91.4	91.6	982.3	96.2
aLDTA	10	88.0 ± 1.9	83.4	88.3	88.6	88.9	89.1

Angles	Number of limbs	Computed tomography measurements (°)					
		Mean ± SD	Minimum	Quartile 1	Median	Quartile 3	Maximum
Norberg	4	118.3 ± 9.9	105.9	114.7	118.7	122.2	130.0
Hauptman B	8	136.4 ± 10.5	120.4	131.2	135.1	140.6	152.1
mLPFA	8	109.7 ± 10.5	94.9	101.5	113.6	116.9	121.2
mLDFA	8	96.9 ± 7.2	90.7	91.6	95.6	98.7	112.6
aMPTA	10	93.4 ± 2.5	90.6	92.1	92.7	93.7	98.9
aLDTA	10	89.9 ± 1.9	86.9	88.5	89.8	91.1	92.7

Abbreviation: SD, standard deviation.

## 4 Discussion

In *T. terrestris* , the Norberg angle was assessed as one of the methods for evaluating the hip joint. This angle is widely used in dogs for the radiographic assessment of hip dysplasia, with animals presenting a deep and congruent acetabulum considered normal when values exceed 105°, whereas values below 105° are indicative of joint laxity (Butler and Gambino, 2017). This parameter has previously been measured radiographically in other clinically normal wild canid species, including the maned wolf ( *Chrysocyon brachyurus* ) (Siqueira et al. 2018) and the crab‐eating fox ( *Cerdocyon thous* ) (Castilho et al. 2021), with mean values of 112.9° (± 1.6°) and 107.57° (± 3.65°), respectively. In normal capybaras ( *Hydrochoerus hydrochaeris* ), the mean value reported was 125.9° (± 4.49°) (Brombini et al. 2018), which is similar to the value found in the present study by radiographic analysis (127.5° ± 1.3°), but not by CT scan (118.3° ± 9.9°). These findings correspond to a qualitatively normal evaluation of the hip joint, characterized by a rounded femoral head well positioned within the acetabulum, as verified on radiographs and CT, a feature also described in an anatomical study of the species (Borges et al. 2018).

To further understand the biomechanics of the hip joint, the femoral inclination angle (angle between the femoral neck and the femoral diaphysis) was also evaluated in the frontal plane. In humans, this angle is associated with the efficiency of the abductor muscles, load distribution across the hip joint and limb length, being classified as coxa valga when greater than 125° and coxa vara when less than 125° (Hamill et al. 2015). In *T. terrestris* , the femoral inclination angle showed a mean value of 133.6° (± 12.78°) by radiography and 136.4° (± 10.5°) by CT, determined using Hauptman's method B, in which anatomical landmarks were easily identified. These values were comparable to those observed in capybaras (139.9° ± 3.59°) evaluated using the same measurement technique on radiographic examination. In humans, individuals with coxa vara exhibit increased loading on the femoral head and reduced loading on the femoral neck (Hamill et al. 2015). It should be noted that, in *T. terrestris* , the femoral neck exhibited a relatively short morphology.

In juvenile *T. terrestris* , both the proximal femoral growth plate and the growth plate of the greater trochanter were identified. One study suggested that the femoral ossification pattern may be an artefact related to femoral shape and femoral neck length; in the case of the Asian tapir ( *T. indicus* ), this pattern was classified as coalescent, meaning that the ossification centres of the femoral head and greater trochanter fuse into a single bony epiphysis (Serrat et al. 2007).

The greater trochanter of the femur in adult *T. terrestris* was well developed and extended beyond the dorsal limit of the femoral head, similar to what is observed in large domestic animals such as horses and cattle (Liebich et al. 2020). As described in anatomical studies, *T. terrestris* presents a lesser trochanter and a third trochanter located on the lateral aspect of the femoral diaphysis, the latter being well developed in the species (Borges et al. 2018). Horses also possess a prominent third trochanter, which serves as the insertion site for the superficial gluteal muscle (Liebich et al. 2020).

One method for evaluating limb alignment is through the determination of angles formed by the anatomical and mechanical axes (Petazzoni and Jaeger 2008; Marques and Varatojo 2021; Michalska‐Foryszewska et al. 2024). In the present study, analysis of femoral mechanical angles in the frontal plane was chosen. Establishing normal reference patterns is essential for identifying deformities that may result in deviations of the physiological axes, whether in the frontal (varus and valgus), sagittal or transverse planes (Marques and Varatojo 2021).

In *T. terrestris* , the mLPFA was 98.9° (± 14.8°) on radiographic examination and 109.7° (± 10.5°) by CT scan, while the mLDFA was 95.3° (± 3.1°) on radiographic examination and 96.9° (± 7.2°) on CT scan. Radiographically, in cadavers of large‐breed domestic dogs, mLPFA values (103.7° ± 5.4°) were reported to be higher than mLDFA values (98.6° ± 2.5°) (Dismukes et al. 2008). In wild carnivores, mean values observed in maned wolf cadavers differed between the right (95.7°) and left (93.8°) limbs for mLPFA, while an mLDFA value of 95.7° was reported (Siqueira et al. 2018). In anaesthetised crab‐eating foxes, mLPFA values of 93.39° ± 4.58° and mLDFA values of 96.75° ± 1.96° have been described (Castilho et al. 2021).

The distal femur in *T. terrestris* exhibited differences between the condyles but presented a symmetrical trochlear groove. Studies have shown that, unlike horses, tapirs do not possess parapatellar fibrocartilage; the medial and lateral trochlear ridges are parallel and of similar proportions, and there is no widening of the medial femoral trochlea (Hermanson and MacFadden 1996; Costa et al. 2022). The conformation of the distal femur, whether asymmetric—when the medial trochlear ridge is larger than the lateral—or symmetric, has been associated with habitat type and locomotor preferences (Janis et al. 2012). In tapirs, there are records indicating that they are capable of trotting, galloping, climbing slopes and swimming proficiently (Padilla and Dowler 1994; Medici et al. 2001; Feldhamer et al. 2015).

The components of the proximal tibial were more clearly identified in adult animals on the craniocaudal view and on CT in the dorsal plan. Using *Tapirus* specimens from different localities, it was observed that the medial condyle is thicker and narrower than the lateral condyle, with both being axially separated by the intercondylar eminence, which is composed of a tall, laminar medial tubercle and a lower, more robust lateral tubercle (Holanda et al. 2012). These characteristics were also identified in the imaging examinations. Another anatomical study demonstrated that the body of the tibia in *T. terrestris* is divided into cranial, caudal, medial and lateral surfaces—the latter presenting slight torsion—with the tibial tuberosity located on the cranial surface (Borges et al. 2021), which is considered an important anatomical landmark (Liebich et al. 2020). This structure was well identified on CT, but visualized on the craniocaudal image as an opacity resulting from border overlap. *T. terrestris* presents a lateral malleolus, which constitutes the distal extremity of the fibula, and a medial malleolus located at the distal extremity of the tibia, forming the medial wall of the cochlea (Borges et al. 2021); both were identified on the craniocaudal radiographic view and CT.

In *T. terrestris* , the aMPTA was 92.2° (± 1.7°) on radiographic examination and 93.4° (± 2.5°) on CT, while the aLDTA was 88.0° (± 1.9°) on radiographic examination and 89.9° (± 1.9°) on CT. In a study with sheep, the aMPTA (89.61° ± 2.5°) and aLDTA (86.64° ± 2.2°), obtained by radiographic examination using the same methodology, were also very close to each other, with no differences between age groups.

The fibula of *T. terrestris* extended laterally and remained separated from the tibia by an interosseous space along its entire length, similar to what is observed in pigs (Liebich et al. 2020). However, although the tibia and fibula are anatomically distinct, one study stated that well‐developed articular facets allowing significant movement are absent between them (Hawkins 2011).

A study revealed that, in *T. terrestris* , only the medial metatarsus articulates with the ectocuneiform (tarsal III) (Earle 1893), a finding that is consistent with verified by CT. The third digit was the largest, as previously described in imaging examinations and anatomical studies (Endo et al. 2019; Borges et al. 2021). A characteristic of the tapirs is the presence of hooves on all digits (Padilla and Dowler 1994; Quse and Fernandes‐Santos 2014). In addition, a study demonstrated that the species' semi‐aquatic locomotion favours adductor–abductor mobility in the phalanges of the most medial and lateral digits of the feet (Endo et al. 2019).

In conclusion, the imaging examinations were complementary, enabling the characterization of morphological features reflecting the species' functional adaptations for cursorial and semi‐aquatic locomotion. The angular measurements evaluated in this study were important for providing quantitative information on joint alignment, stability and limb biomechanics in *T. terrestris* , establishing values that may contribute to anatomical, functional and clinical assessments of the species. However, further studies including a larger number of animals are necessary to establish reliable reference values.

## Funding

This work was supported by Coordenação de Aperfeiçoamento de Pessoal de Nível Superior, Finance Code 001. Financiadora de Estudos e Projetos, agreement no. 01.12.0530.00. Fundação de Amparo à Pesquisa do Estado de São Paulo, process no. 2023/16693‐7.

## Conflicts of Interest

The authors declare no conflicts of interest.

## Data Availability

The data that support the findings of this study are available from the corresponding author upon reasonable request.
